# Impact of Foreign Language Classroom Anxiety on Higher Education Students Academic Success: Mediating Role of Emotional Intelligence and Moderating Influence of Classroom Environment

**DOI:** 10.3389/fpsyg.2022.945062

**Published:** 2022-07-08

**Authors:** Siyuan Han, Yiman Li, Syed Arslan Haider

**Affiliations:** ^1^School of Japanese Culture and Economics, Xi’an International Studies University, Shaanxi, China; ^2^Department of Shipping Navigation and Engineering, Yan’an Vocational and Technical College, Shaanxi, China; ^3^Department of Management, Sunway University, Subang Jaya, Malaysia

**Keywords:** foreign language classroom anxiety, academic success, emotional intelligence communication, class room environment, higher education institution

## Abstract

The current study aimed to investigate the impact of foreign language classroom anxiety (FLCA) on academic success through mediating role of emotional intelligence communication (EIC) and moderating role of class room environment. Due to the disruptive impacts of the COVID-19 pandemic, teaching and learning were moved online nation-wide. The convenient sampling technique was used, for data collection from Chinese university students. There was a total of 615 students that participated in the survey and data gathered in 5 months from November 2021 till March 2022. Covariance-based structural equation modeling (CB-SEM) in SPSS V.25 and AMOS V.22 was used to assess model fitness and hypotheses, as well as construct reliability and validity of the measurement model. The results revealed that FLCA is negatively and significantly influence students’ academic success. Furthermore, EIC as a mediator significantly and positively mediates the relationship between FLCA and academic success. The current study shows that emotional intelligence has the ability to reduce students’ foreign language anxiety and so improve their language skills. Lastly, classroom environment positively and significantly moderates the relationship between FLCA and emotional intelligence communication.

## Introduction

Teaching foreign languages is a difficult career that demands a great deal of energy, imagination, and innovation (Khajavy et al., 2018). The majority of academics in the field of foreign language acquisition agree that learning a foreign language is emotionally charged for students (Imai, 2010; López, 2011; Dörnyei and Ryan, 2015; Mierzwa-Kamińska, 2021). As a result, it is reasonable to conclude that regulating appropriate human emotions throughout the acquisition of a foreign language is an essential component of effective academic communication in students (Oz et al., 2015). Therefore, being emotionally intelligent in a foreign language class is associated with increased academic productivity, positive emotions, and motivation to continue learning the second language (Guslyakova and Guslyakova, 2020). Furthermore, individual differences influence the learning process, which is one of the reasons why foreign language learning is difficult. To put it another way, research findings show that learners’ beliefs, attitudes, expectations, and affective states are important factors that influence the foreign language learning process (Afzali and Izadpanah, 2021). According to the findings of existing research, test anxiety has significant effects on the process as an affective state (Roick and Ringeisen, 2017). The purpose of this study is to investigate the difficulties Chinese students face when learning a foreign language in a classroom setting. It is especially important for Chinese students who have limited actual English communication in their daily lives, as it enables them to create their own speaking opportunities both inside and outside of the classroom, thereby reducing their anxiety and enhancing their proficiency and academic success.

The role of foreign language anxiety in foreign language learning has long been a subject of study in the field of second language acquisition. All of the data points to the impact that foreign language anxiety can have on foreign language learning and performance in a variety of disciplines. Foreign language anxiety, in particular, may have detrimental academic, cognitive, and social consequences for students (Dewaele and MacIntyre, 2016; MacIntyre, 2017). In other words, worry impedes learners’ proficiency progress and undermines their confidence in the foreign language, which in turn enhances anxiety, creating a vicious cycle. Students who are anxious may try to avoid communicating with others (Jin and Dewaele, 2018). One factor in this regard would be individual differences in language ability, willingness to study, attitudes toward the target language community, and available resources all contribute to inter-learner differences in foreign language outcomes (Paul Sun and Jun Zhang, 2022). In addition, emotional experiences in the foreign language classroom that are repeated over time might affect learners’ foreign language learning process (Horwitz, 2017). According to research, foreign language anxiety is caused by a variety of circumstances, including perceived social support, which is one of the possible factors that influence foreign language anxiety. Previous research has pointed to interpersonal differences in personality, temperament, mood, and/or language learning experiences as explanations for why students view social interactions differently (Jin et al., 2017). Furthermore, a positive viewpoint might influence how social bonds are perceived. Students who regularly interpret themselves, others, and life events in a favorable light have a higher opinion of social relationships (Ran et al., 2021). As a result, it is logical to believe that learners who have a good attitude are less likely to have foreign language classroom anxiety (FLCA; Jin and Dewaele, 2018). One of the most prominent purposes of foreign language teaching around the world is to prepare students who are ready and able to speak in another language. The development of communicative competence among language learners has been stressed in foreign and second language instruction, yet proficiency does not always imply a willingness to use the language for meaningful conversation. It is also important to talk and write in order to learn a second language, according to well-known theories like the interaction hypothesis (Khajavy et al., 2018).

The purpose of the current study was to examine the impact of foreign language classroom anxiety on higher education student’s academic success through mediating role of emotional intelligence: moderator class room environment. In the domain of higher education, issues relating to second language learners, particularly in university students with Chinese backgrounds, are significant. Chinese English learners at various educational levels frequently experience anxiety to varying degrees because of their limited use of English in daily life (Lu and Liu, 2015; Liu, 2016; Liu and Xiangming, 2019). Therefore, the majority of learners share their anxieties and apprehensions about studying English as a foreign language as well (Afzali and Izadpanah, 2021; Paul Sun and Jun Zhang, 2022). Anxiety can cause a variety of issues in language acquisition, retention, and production (Pyun and Byon, 2022), affecting student’s grades when compared to other more relaxed classmates. According to Tanir and Özmaden (2018), anxiety is one of the most common psychological symptoms that has a negative impact on university students’ mental health. The term anxiety is defined as a troubling emotion that arises when it appears that a strong desire or impulse will not be fulfilled (Alver et al., 2016). According to Kanero et al. (2022), situation-specific anxiety can be defined as anxiety that is provoked when certain conditions are present including examinations, acting on stage, giving a speech, and/or communicating in a second or foreign language are among the situations described by Horowitz (2001). Anxiety among Chinese students learning a second language is one aspect of the problem. This study’s primary objective was to investigate what generates anxiety during different phases of classroom learning and how anxiety impacts the grades of undergraduate students in China. There is a limited amount of literature on this topic in the Chinese population, and these variables have not been studied combined previously. Therefore, the present study must investigate the effect of foreign language classroom anxiety on the academic performance of higher education students and the significance of emotional intelligence and classroom environment. Second language learners with a greater level of emotional intelligence are better able to control impulses, manage stress, and keep a positive attitude in the face of difficulties and frustrations during the process of acquisition.

## Research Literature

### Broaden and Build Theory

In the field of positive psychology, the Broaden-and-Build theory is the fundamental underpinning theory (Fredrickson, 2003). According to this theory, positive emotions extend people’s thought-action repertoires, which helps them generate social resources, whereas negative emotions restrain people’s thought-action repertoires. Positive emotions, it is claimed, cause learners to acquire more information and build more resources for future language learning. Negative emotions, on the other hand, will reduce learners’ focus and limit the range of possible language input. Interest shifted away from foreign language classroom fear and toward a broader range of feelings such as pleasure, love, pride, hope, humiliation, remorse, and boredom (Dewaele and Li, 2021). Broaden-and-build theory emphasizes the positive predictive effect of positive emotions on academic performance (Fredrickson, 2004). Students will evaluate their own behaviors, causing positive or negative emotional reactions according to the social cognitive theory of self-regulation (Bandura, 1991). When they put in enough effort to learn a foreign language, they tend to positively evaluate their own behaviors, resulting in positive emotional reactions such as foreign language enjoyment. Enjoyment is a positive emotional sense that comes from pushing oneself beyond one’s homeostatic boundaries and doing something challenging (Dewaele and MacIntyre, 2016). The enjoyment of a foreign language can help pupils study more effectively by expanding their cognitive resources. Foreign language enjoyment can also help learners gain positive power, relieve stress, and increase their enthusiasm for foreign language learning (Piniel and Albert, 2018).

### Foreign Language Classroom Anxiety and Academic Success

Anxiety is something that most people experience on a regular basis. It is regarded as one of the most common and enduring human emotions, affecting physiological arousal and cognitive processes (Kanero et al., 2022). Anxiety can be useful when it leads to excitement and enthusiasm, but it can also be harmful when it leads to worry, confusion, fear, and a loss of self-esteem (Karatas et al., 2013). According to Phillips (1992), there are two types of anxiety: state anxiety and trait anxiety. State anxiety is a situation-specific trait anxiety; that is, an individual with state anxiety will have a stable tendency to be anxious, but only in certain situations. Trait anxiety, on the other hand, is a relatively stable tendency to be anxious in a wide range of situations. Consequently, when it comes to learning any language in a classroom setting, individuals face obstacles, which results in language anxiety.

According to Horwitz et al. (1986), language anxiety is a complex of self-perceptions, beliefs, attitudes, and behaviors that develop from the uniqueness of the language learning process in the classroom. The term foreign language classroom anxiety is a mental condition as well as a social construct. To put it another way, internal psychological processes, cognition, and emotional states, as well as the demands of the circumstance and the presence of other people, co-shape it. The feeling of discomfort that language learners feel because they lack the linguistic means to portray themselves genuinely is the main root of foreign language classroom anxiety. Indeed, for some people, presenting themselves to the world through an imperfectly regulated new language is inherently anxiety-provoking (Li and Dewaele, 2021). Due to science, commerce, tourism, technology, and other factors, learning a foreign language has become increasingly important for many individuals all over the world. But the process of learning English as a second language is affected by many psychological factors, such as self-esteem, self-efficacy, motivation, and attitudes, as well as some linguistic factors, such as language anxiety, cultural background, and learning style. Horwitz (2017) said that students suffer from anxiety when learning English as a foreign language. Several students experience significant levels of stress when learning a foreign language, according to Riasati (2011), who urged that English language teachers be aware of language anxiety in the classroom and identify ways to simplify the language learning process.

Horwitz et al. (1986) identified three categories of foreign language anxiety: Firstly, communicative apprehension refers to a learner’s level of concern or fear in relation to actual or anticipated communication with others. Researchers have become increasingly interested in the elements that influence English as a foreign language learners’ performance in recent years. According to Paul Sun and Jun Zhang (2022), when compared to other courses, oral learners have a higher level of anxiety. Anxiety over speaking in public is a typical problem among English language students. Another study discovered that 20% of participants had anxiety about performing in front of an audience (Mohamad et al., 2009). In addition, he found that one out of every five students who had oral performance anxiety had a negative effect on their oral performance and grades. Secondly, test anxiety is the inclination to evaluate one’s performance in an evaluating scenario. When students’ performance on previous examinations has been poor, they develop test anxiety. As a result, the students have a negative perspective on tests and have incorrect perceptions when evaluating circumstances. Unconsciously, this bad impression is passed on to the English class. Similarly, students may have wrong perceptions of language learning. They may consider any poor test result a failure. According to Roick and Ringeisen (2017), test anxiety can have a greater impact on weak students’ performance than on students with higher competence levels and more anxiety in evaluative settings. According to a study, test anxiety and foreign language anxiety had negative statistical effects on students’ examination grades (Mohamed Khalifa Gawi, 2020). Additionally, test anxiety has a noticeable impact on classroom discourse and student performance. Test anxiety is one of three components of foreign language anxiety generated by the dread of failing examinations (Horwitz, 2017). Thirdly, Horwitz et al. (1986) describe fear of negative evaluation as the fear of others’ assessment and evaluation. According to Gardner and MacIntyre (1993), fear of unfavorable appraisal is directly linked to fear of communication. When pupils are unsure of what they are saying, they are afraid of being judged negatively and doubt their ability to provide positive results. The fear of being judged negatively is one aspect of the anxiety associated with learning a foreign language. It is also linked to a negative interpretation of social feedback and appraisal. It is a sense of failure, as well as a lot of attention from other people’s opinions. Students’ performance during evaluations or social activities such as job interviews or in English lessons when it comes to speaking can reveal their fear of bad criticism (Afzali and Izadpanah, 2021). Fear of negative assessment, test anxiety, and communication apprehension were identified as important theoretical frameworks for illustrating the foreign language by Horwitz et al. (1986).

Previous research has looked into the impact of anxiety on English as a Second Language learning (Liu and Xiangming, 2019). The findings of the studies demonstrated the harmful impact of anxiety on the learning of English. A high level of anxiety can lead to a variety of issues, including disappointing students and poor performance. Learners with high anxiety levels frequently perform poorly, have low achievement, and are nervous when it comes to learning. Students who perform poorly in English language classes and assessments feel high levels of anxiety (Mohamed Khalifa Gawi, 2020). To alleviate student tension, English language teachers should create a pleasant atmosphere in the classroom. On the other hand, students’ anxiety goes up when teachers are too serious or strict in the classroom (Razak et al., 2017).

Prior research revealed a weak link between foreign language speaking anxiety and participants’ language learning outcomes (Al-Khotaba et al., 2019). According to the findings of Alaleh (2018), the participants had a moderate level of reading and language anxiety. It also found that the primary drivers of foreign language reading anxiety include problems understanding new words’ meanings, pronunciation, difficulties reading extensive texts, and the fear of making mistakes. It should also be noted that examination anxiety is a hindrance to students’ successful performance (Roick and Ringeisen, 2017). Many researchers have studied the relationship between foreign language anxiety and students’ academic success, and it has been concluded that foreign language classroom anxiety has a negative impact on student performance (Razak et al., 2017). Therefore, we have developed the following hypothesis:

*H1*: Foreign language classroom anxiety has a negative impact on academic Success.

### Foreign Language Classroom Anxiety and Emotional Intelligence Communication

Emotional intelligence is defined as the ability to notice emotions, access and generate emotions to aid thought, comprehend emotions and emotional knowledge, and reflectively control emotions to enhance emotional and intellectual development (Mayer and Salovey, 1997). It integrates all possible feelings and emotional skills into a unified framework as a theoretical construct. As a result, it is thought to enable an individual to recognize and regulate unpleasant emotions as well as to develop and use positive emotions to aid thinking. MacIntyre (2002) said about language learning, in some ways, learning a language can cause a lot of strong feelings.

Negative emotions like worry, fear, tension, and wrath, in particular, might jeopardize a learner’s ideal learning potential and significantly diminish their language learning ability (Ran et al., 2022). Positive emotions, on the other hand, such as self-esteem, empathy, motivation, and enjoyment, can place learners in the best possible state for language learning and considerably facilitate the process. Second language learners with a greater degree of emotional intelligence are better able to regulate impulses, manage stress, and keep a good attitude in the face of challenges and frustrations. In short, emotional intelligence claims to be able to predict how learners will react to the demands of distinct second language learning and usage settings, which are critical for successful second language acquisition. Two distinct models have emerged from research on the measurement of emotional intelligence (Zeb et al., 2021). The ability model of emotional intelligence uses a purely cognitive metric to elicit maximum performance on particular emotional information processing activities from test takers. The trait model of emotional intelligence, on the other hand, is concerned with behavioral dispositions and self-perceived abilities as judged by self-reports that heavily reflect personality factors. In trait emotional intelligence theory, emotional intelligence is viewed as a collection of emotion-related self-perceptions and dispositions. This theory is in line with not only the most common theories of personality, but also with most of the evidence from a number of different areas, such as life satisfaction, ruminating, and coping methods (Shao et al., 2013).

The nature of foreign language anxiety (FLA) is a critical question based on the substantial research that documents the importance of EIC for learning in general. Individuals with high emotional intelligence feel they can control their emotional reactions over time, manage stress, and assert themselves. They are also more likely to be confident in their capacity to communicate well in a foreign language, which reduces their risk of foreign language acquisition (Dewaele et al., 2008). These kinds of theoretical and empirical discoveries are especially important for educational systems with huge numbers of foreign language learners, such as China, which has the world’s largest number of English-speaking foreign language students. Even when they are high performers, most of these students have few opportunities to speak English outside of the classroom and are often hesitant to use the language in public or naturalistic contexts. As a result, there is a pressing need to address their emotional needs and feelings while learning a foreign language, for the obvious reason that knowing how to reduce anxiety on the part of students themselves may improve learners’ ability to become successful language users and language learners (Shao et al., 2013).

Previous research has found that language learners who have a higher level of trait emotional intelligence are less worried during their language learning process (Li, 2020). In a previous study, trait emotional intelligence, which includes components like emotion regulation, stress management, and assertiveness, was found to have a significant negative connection with anxiety levels in the English classroom among Chinese university students. Higher-trait emotional intelligence individuals are thought to be better able to control their own emotions and gauge the emotional reactions of others, allowing for more fluid interpersonal relationships and reduced anxiety levels. The discovery that students with high levels of emotional intelligence have lower levels of foreign language classroom anxiety (Jin and Dewaele, 2018). On the basis of the above literature, we have developed the following hypothesis:

*H2*: Foreign language classroom anxiety is negatively associated with emotional intelligence communication.

### Emotional Intelligence Communication and Academic Success

Several studies have found that emotional intelligence positively predicts academic success in a variety of educational settings (Li, 2020). The correlations between foreign language classroom anxiety and academic achievement, as well as between emotional intelligence communication (EIC) and academic success, found in a Chinese university English foreign language context, suggest that emotionally competent students are more optimistic about their English proficiency, have more self-confidence, and have better actual performance (Shao et al., 2013). The majority of studies in the realm of education have focused on the relationship between EIC and academic performance. Previous research has shown that EIC can predict pupils’ academic performance in future (Ran et al., 2022). In another study, EIC was revealed to be a strong predictor of students’ academic achievement. Overall, past research has found that trait EI has a correlational or facilitative effect on a variety of significant life outcomes (Chen and Zhang, 2020). Therefore, the abovementioned literature leads to the following hypothesis:

*H3*: Emotional intelligence communication is positively associated with academic success.

### Mediating Role of Emotional Intelligence Communication Between Foreign Language Classroom Anxiety and Academic Success

According to a prior study, the mediated impact arises when students with higher emotional intelligence participate in more positive interpersonal activities, such as competent communication (Ran et al., 2022). They will also have more influence over the communication behaviors of other team members. Students with greater emotional intelligence will initiate reciprocal contact inside the group, which will increase their emotions of attraction and belonging to the team (Troth et al., 2012). The results demonstrated that trait emotional intelligence was positively connected to foreign language English scores, implying that learners with a higher trait emotional intelligence were more likely to enjoy foreign language learning. According to the ability EI model, which includes the ability to generate positive thought-facilitating emotions, is based on a substantial association between students’ trait emotional intelligence and foreign language English scores, which has been conceptualized as a thought-broadening positive emotion (Li, 2020). Previous research has discovered that emotional intelligence can considerably alleviate and regulate negative emotions such as foreign language acquisition, which has an indirect positive impact on foreign language success (Yu, 2021). The above literature leads us to hypothesize that:

*H4*: Emotional intelligence communication mediates the relation between foreign language classroom anxiety and academic success.

### Moderating Role of Classroom Environment Between Foreign Language Classroom Anxiety and Emotional Intelligence Communication

Classroom environment refers to the atmosphere, ambience, tone, or climate that pervades classroom settings (Dorman et al., 2006). It is crucial to learning because it influences how students think, feel, and act in the classroom (Jennings and Greenberg, 2009). Student cohesiveness, teacher support, self-involvement, investigation, task orientation, cooperation, and equity are all characteristics of a positive classroom environment (Li and Dewaele, 2021). The relationship between the classroom environment and students’ cognitive and affective outcomes has long been the focus of general education research. Negative classroom environments are linked to negative feelings, inattention, and lack of engagement in learning activities, whereas positive classroom environments are linked to positive feelings, heightened attention, motivation, and engagement in the learning environment (Yafi et al., 2021). The study of second language acquisition has gone through a similar transformation. As foreign language students’ high levels of classroom anxiety may be due in part to their cognitive and emotional assessments of the classroom environment, as a result, anxiety has both internal and social dimensions. In a previous study, perceived student engagement and teacher support were found to be negatively related to foreign language classroom anxiety scores, but there was a positive relationship between perceived task orientation and anxiety in Spanish students at both levels (Jin and Dewaele, 2018). Students with a higher positive orientation are more open to pleasant moments, more sensitive to signals of reward from teachers or peers, and less concerned about setbacks in learning. They can also recover from frustration faster. All of this adds to higher positive orientation students’ subjective well-being, as measured by reduced anxiety, and, as a result, optimal foreign language classroom performance. Learners who think less favorably, on the other hand, are more prone to noticing negative features of situations or people, and are more likely to misinterpret something nice or neutral as bad (Jin and Dewaele, 2018).

**Figure 1:**
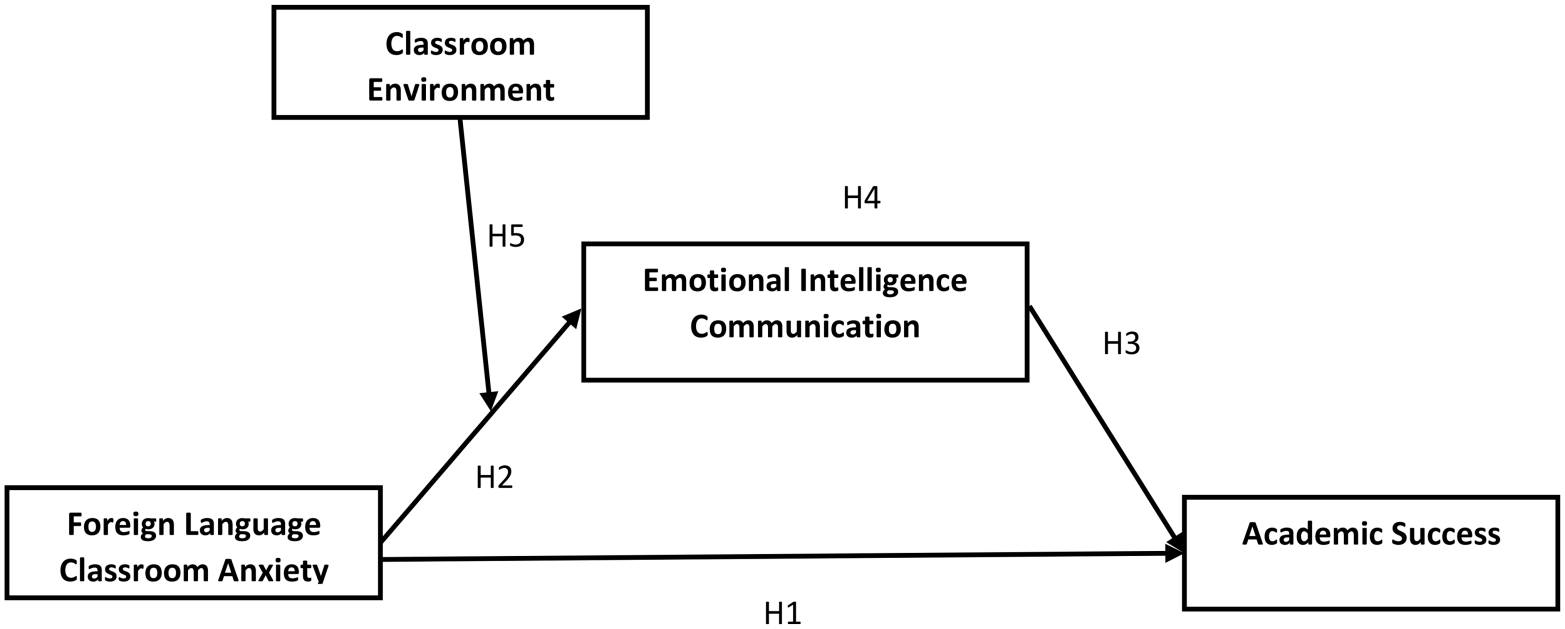
Research model.

Previous research has suggested that contextual and situational factors may also play a role in the level of test anxiety. Environmental and situational variables, it was discovered, had significant differential effects on high-and low-test-anxious students, masking learning performance (Khajavy et al., 2018). Another study attempted to determine a rough estimate of a language learner’s aptitude in oral communicative exchange. The findings revealed that the examiner’s flexibility and easygoing demeanor, as well as a comfortable seating arrangement that does not put the examiner and learner in direct opposition, can help to alleviate some stress; nonetheless, reducing some sources of student anxiety can be challenging (Roick and Ringeisen, 2017). The present study confirms that the classroom environment plays a moderating role FLCA and EIC (see Figure 1). Therefore, Hypothesis 5 is constructed as shown below:

*H5*: Moderating role of classroom environment between foreign language classroom anxiety and emotional intelligence communication.

### Research Methodology

Due to the disruptive impacts of the COVID-19 pandemic, teaching and learning were moved online nation-wide. The convenient sampling technique was used (Taherdoost, 2016), for data collection from Chinese university students. The first author specifically contacted the head of department of public and private universities in China. The head of department agreed to help with data collecting and sent the online survey to university students through a WeChat (a popular social media app) group. The data gathered in five months from November 2021 till March 2022. Those students who want to participate further shared the questionnaire to their class WeChat group. Students were informed of the survey’s nature, purpose, estimated completion time, anonymity, and their freedom to refuse the invitation or withdraw at any time. An aggregate of 700 surveys were distributed and 640 were returned. After the deletion of 25 incomplete responses, a total of 615 surveys were selected, yielding a response rate of 87.85%. Harman’s single factor test is performed after data collection to find common method variance. The result of the extraction sums of squared loading is 24.44% of variance, which is less than 50%, indicating that there is no common method bias issue in the data (Tehseen et al., 2017). There was a total of 615 students that participated in the survey. There were 368 male (59.8 percent) and 247 females (40.2 percent). The majority students were 22 to 35 years old and have Bachelors and Master’s degree holders. The students were enrolled in English and Chinese language courses, these two languages were not their main language. Although, the Ministry of Education of the People’s Republic of China issued the same English textbook and curriculum for all students.

### Instruments

Data were gathered through questionnaires, and all construct items were adapted from existing sources. A pilot study was conducted to confirm that the instrument is valid before conducting a large-scale investigation (Van Teijlingen et al., 2001). All questions were evaluated using a five-point Likert scale: (1) Strongly Disagree, (2) Disagree, (3) Neutral, (4) Agree, and (5) strongly Agree. This instrument is suitable for data collection since it helps to gather information quantitatively in an efficient and easy manner. The foreign language classroom anxiety (FLCA) scale based on 33 items was used to assess foreign language anxiety (Horwitz et al., 1986). It was first designed to assess foreign language specific anxiety. It is usually thought to have a one-factor structure that measures three types of anxiety: communicative apprehension, test anxiety, and fear of negative evaluation (Horwitz, 2017). Fraser et al. (1986) developed a scale with 56-items that was used to measure moderating variable classroom environment (CE). Davies et al. (2010) designed a 10-item scale that was used in the process of measuring mediating variable Emotional Intelligence Communication (EIC). Lastly, the academic success (AS) was evaluated with the use of a 50-item scale that was developed by Prevatt et al. (2011).

## Results

### Measurement Model

A measuring model and a structural model were both used by Anderson and Gerbing (1988) in order to put our theory to the test. Covariance-based structural equation modeling (CB-SEM) in SPSS V.25 and AMOS V.22 was used to assess model fitness and hypotheses, as well as construct reliability and validity of the measurement model. The technique of measurement indicated the validity and reliability of the constructs. The suggested models were tested using a maximum likelihood method to confirmatory factor analysis (CFA; Brown and Moore, 2012). Based on an evaluation of Cronbach’s Alpha, the scale’s reliability was determined. Cronbach’s alpha values ranged from 0.563 to 0.882, indicating good reliability, according to Taber (2018). In addition, convergent and discriminant validity were evaluated using factor loadings, composite reliability (CR), and average extracted variance (AVE). Factor loadings that are more than 0.70 are considered substantial in the majority of cases. It is only advisable to remove items with factor loadings ranging from 0.40 to 0.70 if doing so would result in an improved CR or AVE score (Ong and Puteh, 2017). According to Table 1, which shows that all estimates of CR, and AVE exceeded the set cutoff requirements, the measurement model is valid and convergent. Since the correlation between each variable is greater than its square root, we may conclude that our model is appropriate. The results of the measurement model show that it is very reliable and valid in this manner (see Table 1).

**Table 1:** Measurement model.

Construct	Cronbach’s Alpha	CR	AVE	Source
FLCA	0.834	0.878	0.548	Horwitz et al., 1986
Academic Success	0.795	0.858	0.548	Prevatt et al., 2011
Classroom Environment	0.866	0.875	0.367	Fraser et al., 1986
EIC	0.822	0.875	0.585	Davies et al., 2010

FLCA, Foreign language classroom anxiety; AS, Academic success; CE, Classroom environment; EIC, Emotional intelligence communication; CR, Composite Reliability; AVE, Average variance extracted.

### Model Fit Analysis

In our examination of the theoretical structure, we used the methodology proposed by Byrne (2001) and made use of several goodness of fit criteria. According to the findings of the structural model study, the proposed model has a satisfactory match with values that are either more than or equal to 0.90 percent. Incremental fit index (IFI), Comparative fit Index (CFI), Goodness of Fit Index (GFI), Adjusted Goodness of Fit Index (AGFI), root mean square error of approximation (RMSEA), and chi-square fit statistics/degree of freedom (CMIN/dfZ) were used to determine whether or not the tested model should be accepted or rejected. We considered values of CMIN/df less than 5.00 as acceptable in order to comply with the guideline given by Tao et al. (2021). Given that CMIN was dependent on sample size, we found that this was an appropriate threshold. When the RMSEA was less than or equal to. 08, GFI, IFI, and CFI values that were more than or equal to. 95 were considered to be good fits in the available research. It is abundantly obvious that the provided hypothetical model has a strong overall data fit, and its application concerns the estimation of foreign language classroom anxiety, academic success, classroom environment, and emotional intelligence communication. The findings of this structural model revealed good fit (IFI = 0.93, CFI = 0.93, GF1 = 0.94, AGFI = 0.91, the CMIN/df = 2.40, and the RMSEA = 0.039), as given in Table 2.

**Table 2:** The analysis of model fit (metric invariance).

Measurement models	IFI	CFI	GFI	AGFI	CMIN/df	RMSEA
Threshold values	> 0.9	> 0.95	> 0.95	> 0.8	< 3	0.05–0.1
Configural invariance (baseline model)	0.865	0.868	0.845	0.765	4.321	0.059
Metric invariance	0.935	0.935	0.940	0.915	2.40	0.039

### Correlation Analyses

A correlation study was performed to establish the relationships between variables (Cohen et al., 2014). Pearson correlation determines the degree and type of a relationship using a correlation between −0.1 and 0.1. Positive sign indicates variables going in the same direction, whereas negative sign indicates variables moving in the opposite direction. Furthermore, the “r” value indicates the link’s strength.

Table 3 indicates depicts information related to the correlation between variables. Correlation table shows that independent variable foreign language classroom anxiety has significant and negative correlation with academic success (*r* = −0.392, *p* < 0.05), emotional intelligence communication (*r* = −0.552, *p* < 0.05), and classroom environment (r = −0.443, *p* < 0.05). Furthermore, the mediating variable emotional intelligence communication correlation with academic success (*r* = 0.755, *p* < 0.05), and classroom environment (*r* = 0.281, *p* < 0.05), were also positive and significant. Lastly, the classroom environment the moderating variable also positively and significantly correlated with academic success (*r* = 0.146, *p* < 0.05).

**Table 3:** Correlation analyses.

Constructs	AS	CE	EI	FLCA
Academic Success	1			
Classroom Environment	0.146^**^	1		
EIC	0.755^**^	0.281^**^	1	
FLCA	−0.392^**^	−0.552^**^	−0.443^**^	1

FLCA, Foreign language classroom anxiety; AS, Academic success; CE, Classroom environment; EIC, Emotional intelligence communication; CR, Composite Reliability; AVE, Average variance extracted; *N* = 615.

** Correlation is significant at the 0.01 level; ^*^Correlation is significant at the 0.05 level; ^***^Correlation is significant at the 0.001 level (2-tailed), ^**^p < 0.01.

### Mediation and Moderation Analyses

For analysis of mediation and moderation, Hayes and Preacher (2014) methods were used. Model 4 is used for mediation analysis, whereas model 7 is used for moderation mediation analysis. Regression analysis is a method that evaluates the statistical relationship between two or more variables (association). The degree to which a result variable is reliant on the predictor variable is shown through regression analysis. It explains how the estimate of a measure variable varies when a variation occurs in one or more independent variables. As a result, it reveals the causal link between variables, while correlation analysis only describes the association between variables. The regression process is carried out using a variety of methods (for example, Baron and Kenny, 1986), however for the convenience and suitability of the study, Hayes and Preacher (2014) process approach is used for investigation.

According to Preacher and Hayes (2008), methodology is antiquated since it requires a condition of absolute causality for intervening, which some experts believe is unnecessary and even a hindrance in the process for testing genuine effect (Hayes and Preacher, 2014). According to these researchers, the indirect effect through mediation is also possible even if no indications of direct influence between predictor and outcome components were detected (Hayes, 2012). Furthermore, because information in sociology is constantly vulnerable because of the circumstance, nature, and setting of respondents, the bootstrapping procedure for intercession in Hayes (2012) process technique builds the amiability of satisfactory outcomes because the example is divided into numerous small odds and ends and analysis is kept running on those smaller measured subsamples.

The results of hypothesis testing are shown in Table 4. First, the direct impact H1, H2, and H3 were investigated that “foreign language classroom anxiety is negatively associated to academic success.” The results show that there is a negative and significant association between FLCA and AS (β =−0.070, *p* < 0.05). The value of β demonstrates the percentage change, illustrating that a one-unit change in FLCA results in a −0.070 unit change in AS. The findings show that about −7% of the change on the dependent variable is observed, and a value of p of 0.05 indicates a greater degree of significance, providing solid reasons to accept hypothesis H1. Also, support the relationship between FLCA and EIC (β =−0.445, *p* < 0.001), which indicated that FLCA decreases EIC by −44.5 percent. Finally, the relationship between emotional intelligence and academic success is positive significant (β =0.727, *p* < 0.001), and emotional intelligence bring 72.7 percent change in academic success

**Table 4:** Mediation and moderation analysis.

Hypotheses	Relationship among construct	*β*	Mean	SD	*T*-value	*P*-value	LLCI 2.5%	ULCI 97.5%	Remarks
	Direct Effect								
H1	FLCA → AS	−0.070	−0.072	0.030	2.349	0.019^*^	0.013	0.130	Supported
H2	FLCA → EIC	−0.445	−0.441	0.042	10.583	0.000^***^	0.357	0.522	Supported
H3	EIC → AS	0.727	0.727	0.022	32.718	0.000^***^	0.682	0.770	Supported
	Mediating Effect								
H4	FLCA → EIC → AS	0.323	0.321	0.032	10.035	0.000^***^	0.258	0.384	
	Moderating Effect								
H5	FLCA ^*^ CE → EIC	0.197	0.190	0.036	5.522	0.000^***^	0.120	0.259	Supported

FLCA, Foreign language classroom anxiety; AS, Academic success; CE, Classroom environment; EIX, Emotional intelligence communication; CR, Composite Reliability; AVE, Average variance extracted; LLCI, Lower limit confidence interval; ULCI, Upper limit confidence interval.

* *p* < 0.05 and ^***^p < 0.001.

According to the results FLCA indirect impact on AS through mediator emotional intelligence is positive and significant (β =0.323, *p* < 0.001). Table 4 reveals the bootstrapping results of Lower Limit Confidence Interval (LLCI) = 0.258 and Upper Limit Confidence Interval (ULCI) = 0.384, without having any zero between both limits, which clarifies that the results are significant. Lastly, the results indicated that classroom environment positively and significantly moderate the relationship between FLCA and EIC (β =0.197, *p* < 0.001). The LLCI is 0.120 and ULCI is 0.259, which shows that there is no zero in the 95% bootstrap confidence interval. Hence, the Hypothesis H4 and H5 were accepted.

## Discussion

The purpose of this study was to examine the impact of foreign language classroom anxiety on academic success among Chinese students, by looking into the role of emotional intelligence communication as a mediator and the classroom environment as a moderator. As foreign language anxiety is said to be the most powerful predictor of foreign language performance among affective components. The learner develops attitudes and feelings toward learning a new language skills scenario after a few experiences within the foreign language setting. If these encounters are negative, foreign language anxiety may arise; if these negative experiences continue, foreign language anxiety becomes a regular occurrence, and the learner becomes anxious and performs poorly. Anxiety and failure expectations are heightened by poor performance and bad emotional reactions, and the following anxiety is a reaction to this perceived threat (Razak et al., 2017). Foreign language lessons have been found to be the most anxiety-inducing classes (Tuncer and Dogan, 2015). The role of emotional intelligence is becoming more widely recognized as a critical characteristic that can influence not only the quality of people’s lives but also their chances of success in any endeavor. This is perhaps truer in the field of language teaching and learning than in many other areas. This is due to the fact that the language we use is so strongly associated with who we are and who we strive to be. As a result, we believe that emotional intelligence and classroom environment is the cornerstone, the most basic components, and the very foundation of foreign language learning.

The following were the hypotheses of the study: Firstly, foreign language classroom anxiety has a negative impact on academic success. The results of the present study were consistent with previous literature. In previous studies, three recent meta-analyses found that foreign language classroom anxiety and achievement measures have substantial negative relationships (Li and Dewaele, 2021). Previous research has indicated that as a learner’s academic performance deteriorates, so does his or her anxiety over certain academic assignments. Similarly, it is commonly established that a worrier learner would struggle academically. According to several research reports, there is a negative association between foreign language anxiety and success in language learning or language competency (Afzali and Izadpanah, 2021), and foreign language anxiety has a detrimental impact on academic progress in language acquisition. A substantial speaking anxiety element has also been documented in the literature as a common component of foreign language classroom anxiety. The results of comparable investigations revealed that anxiety impasses language development. Furthermore, another study found that the students’ anxiety levels fluctuated and grew over time during their English prep instruction and that this fluctuation was a strong predictor of their academic achievement (Tuncer and Dogan, 2015).

Secondly, foreign language classroom anxiety is negatively associated with emotional intelligence communication. According to research, social and emotional abilities have been linked to success in a variety of areas, including successful teaching, student learning, excellent relationships, and academic performance (Jin et al., 2017). Many studies show that emotional intelligence is beneficial in the workplace and in school, and that it improves interviewing, cognitive tasks, and contextual performance (Zeb et al., 2021). In a previous study, metacognitive, affective, and social learning techniques were found to contribute positively to English language proficiency. Another study discovered a relatively high positive association between EI and writing ability (Guslyakova and Guslyakova, 2020).

Thirdly, emotional intelligence communication is positively associated with academic success. The findings of the study were consistent with the prior literature. A study discovered that there was a rather high positive link between EIC and writing ability (Guslyakova and Guslyakova, 2020). The findings revealed that emotional intelligence and linguistic achievement are inextricably linked. Previous studies have also shown that emotional intelligence has a positive impact on academic attainment (Ran et al., 2021). In another study, the relationship between emotional intelligence and English language learning was investigated (Paul Sun and Jun Zhang, 2022). The results showed that the two variables were substantially connected. Emotional intelligence has been proven to have a significant impact on pupils’ linguistic abilities. As a result, it may be argued that emotional intelligence has the potential to improve learning in general and educational goals in particular (Dastgoshadeh and Javanmardi, 2021).

Fourthly, emotional intelligence communication mediates the relationship between foreign language classroom anxiety and academic success. Many of the findings of this study, as previously noted, are similar to past research in that it discovered a series of significant correlations between students’ emotional intelligence, foreign language acquisition, and English achievement (Shao et al., 2013). This study promotes student views of social cohesion by increasing understanding of how EIC is resourced and utilized within students through the promotion of competent communication procedures. The fact that emotional intelligence and communication ability have a mediating influence has practical implications (Troth et al., 2012). When calculating student allocation configurations, the level of a student’s EIC may be a relevant factor to consider. While, there is some controversy about whether EIC can be taught, there is substantial evidence that individuals can be taught communication skills and communication norms. Students who receive communication skills training early in their university careers may be better equipped to engage in teamwork and have a more favorable experience. This emphasizes that improving communication and emotional skills should be a priority in order to maximize performance opportunities (Troth et al., 2012).

Lastly, the moderating role of the classroom environment between foreign language classroom anxiety and emotional intelligence communication. The relationship between the classroom environment and student emotions has been studied by a number of second language acquisition academics. A study revealed a positive association between classroom environment and enjoyment as well as a negative relationship between classroom environment and anxiety. In addition, Li and Dewaele (2021) discovered that classroom environment and trait emotional intelligence together predicted both foreign language enjoyment and foreign language classroom anxiety in a Chinese-English foreign language environment. The conceptual assumptions and actual findings support the notion that the classroom environment and foreign language emotions are linked (Li and Dewaele, 2021). Furthermore, the classroom environment influences the association between foreign language anxiety and academic success as well as the relationship between foreign language anxiety and emotional intelligence communication. As previously noted, many of the findings of this study are consistent with past research in that it discovered a number of significant correlations between students’ emotional intelligence, foreign language acquisition, academic success, and classroom environment (Zeb et al., 2021). In such circumstances, it is reasonable to predict that students with higher emotional intelligence would have less or no language anxiety and would achieve better levels of language proficiency (Li and Dewaele, 2021).

### Theoretical Implications

This study theoretically supports the literature in various ways. First, previous studies have not examined foreign language classroom anxiety with these variables together. The current study’s findings revealed that emotional intelligence and classroom environment play an important role in foreign language anxiety among Chinese students. This study also employs broaden and built theory of positive psychology, emotional intelligence model and social cognitive theory to investigate the conceptualized path. Moreover, the role of emotional intelligence communication as a mediator between foreign language classroom anxiety and academic success and class room environment as a moderator between FLCA and emotional intelligence communication are also new contributions to this study.

### Practical Implications

The following are the study implications: First, teachers should attempt to develop a classroom culture in which language mistakes are accepted as a natural part of the learning process. Prioritize instilling the attitude that mistakes are chances for learning. Second, teachers can employ cooperative learning to complete tasks with students in small groups. Cooperative learning encourages peer collaboration, intentional communication, and interaction with real-world literature. Third, effective praise and feedback should focus on the effort and care that the student put into the task, on the advances in knowledge or abilities, rather than encouraging students to compare themselves to others. Fourth, criticism should not be used to scold students or make them feel bad about themselves. Instead, it should be used to teach students how they can improve.

It is also recommended that the classroom climate be warm, supportive, and motivating. Learners should understand that making a mistake is not fatal and that they are not alone in making mistakes when learning a foreign language. Many students are happy to hear that they are not the only ones who are worried about learning and utilizing a foreign language, as Horwitz (2017) pointed out. Thus, it is critical to handle anxiety-inducing situations with caution. Although it is neither practicable nor useful to totally avoid all anxiety-inducing circumstances, teachers and course designers should create teaching activities that can assist students in reducing their anxiety. It is only possible when foreign language teachers are aware of their students’ difficulties and anxiety-provoking situations, as well as have the skills to deal with them. Teachers should also provide more understandable input to their students. Teachers can do this by speaking at a slower pace in class. They may occasionally switch from the target language to the learner’s native language if they are having difficulty understanding the foreign language.

Roick and Ringeisen (2017) discovered that a teacher’s personality and interactions with students can minimize students’ language anxiety by creating a calm environment. To reduce students’ anxiety, Dewaele and Li (2021) claimed that teachers should utilize an effective teaching strategy that develops respect for students’ sentiments. Furthermore, teachers should employ games to help students relax. They should also aim to replace previous teaching methods, which mostly focused on teachers, with approaches that emphasize the role of students, i.e., a student-centered approach. Finally, these significant pedagogical consequences can assist instructors in overcoming classroom anxiety language, such as teacher trainers emphasizing the value of teacher passion to trainee teachers and strategies to convey it clearly, verbally, and nonverbally. These tactics, when combined with the skillful use of humor, care, and sympathy for students, can result in positive emotional contagion, resulting in increased student engagement, progress, and well-being for both students and teachers. Emotional intelligence should be a part of all educational activities across the board.

### Limitations of the Study

The study had certain drawbacks. This study adopted a purely quantitative method to investigate students’ emotional intelligence, classroom environment, and foreign language acquisition, which may in some ways fail to reveal the learners’ real situations and experiences. Some students may be unable to fully comprehend and appropriately assess their emotional intelligence and foreign language skills, undermining the validity of the research. It would be able to get a more thorough picture of these concerns by combining qualitative approaches like observation, interviews, and reflective journals. Teachers can understand how well students are aware of their emotions in English class and how they use emotional intelligence to manage their anxiety and language acquisition, for example, by paying close attention in the classroom. More research will be done in this area, with different variables based on Chinese undergraduate difficulties.

### Suggestions for Future Researches

The following are the suggestions for future researchers: Future research may employ a mixed-method approach that includes both quantitative and qualitative methods. It is suggested that in future studies, the elements that positively affect test anxiety be further investigated. Furthermore, follow-up studies could expand the number of participants to allow for a more powerful analysis as well as incorporate a variety of test anxiety measures. Therefore, in future, higher education institutions should focus on advanced and instructional resources in the classroom, such as posters, flashcards, comfortable lighting, and music, to produce a favorable learning environment that allows students to relax and enjoy themselves. Teachers and examiners should also get training to learn more about how test anxiety affects the learning process. Studies have shown that test anxiety has a big effect on students’ achievement, performance, competence, and language skills.

## Data Availability Statement

The raw data supporting the conclusions of this article will be made available by the authors, without undue reservation.

## Author Contributions

All authors listed have made a substantial, direct, and intellectual contribution to the work and approved it for publication.

## Funding

This work was supported by Chinese Academic Foreign Translation Project “Sociology and China’s Great Changes” of the National Social Science Fund of China in 2020 (Project No. 20WSHB006) and Xi’an International Studies University’s first New Liberal Arts Research and Reform Practice Project “Research on the Construction of Translation Talent Training Mode from the Perspective of International Communication” (Project No. XWK21YB04).

## Conflict of Interest

The authors declare that the research was conducted in the absence of any commercial or financial relationships that could be construed as a potential conflict of interest.

## Publisher’s Note

All claims expressed in this article are solely those of the authors and do not necessarily represent those of their affiliated organizations, or those of the publisher, the editors and the reviewers. Any product that may be evaluated in this article, or claim that may be made by its manufacturer, is not guaranteed or endorsed by the publisher.
